# Level and determinants of county health system technical efficiency in Kenya: two stage data envelopment analysis

**DOI:** 10.1186/s12962-021-00332-1

**Published:** 2021-12-06

**Authors:** Edwine Barasa, Anita Musiega, Kara Hanson, Lizah Nyawira, Andrew Mulwa, Sassy Molyneux, Isabel Maina, Benjamin Tsofa, Charles Normand, Julie Jemutai

**Affiliations:** 1 Health Economics Research Unit, KEMRI-Wellcome Trust Research Programme, Nairobi, Kenya; 2 Centre for Tropical Medicine and Global Health, Nuffield Department of Medicine, University of Oxford, Oxford, UK; 3 Faculty of Public Health and Policy, London School of Hygiene and Tropical Medicine, London, UK; 4 County Department of Health, Makueni County Government, Makueni, Kenya; 5 Health Systems and Research Ethics Department, KEMRI-Wellcome Trust Research Programme, Kilifi, Kenya; 6 Health Financing Department, Ministry of Health, Nairobi, Kenya; 7 Centre for Health Policy and Management, Trinity College, The University of Dublin, Dublin, Ireland; 8 Cicely Saunders Institute, Kings College London, London, England

**Keywords:** Efficiency, Decentralization, Devolution, DEA, Kenya

## Abstract

**Background:**

Improving health system efficiency is a key strategy to increase health system performance and accelerate progress towards Universal Health Coverage. In 2013, Kenya transitioned into a devolved system of government granting county governments autonomy over budgets and priorities. We assessed the level and determinants of technical efficiency of the 47 county health systems in Kenya.

**Methods:**

We carried out a two-stage data envelopment analysis (DEA) using Simar and Wilson’s double bootstrap method using data from all the 47 counties in Kenya. In the first stage, we derived the bootstrapped DEA scores using an output orientation. We used three input variables (Public county health expenditure, Private county health expenditure, number of healthcare facilities), and one outcome variable (Disability Adjusted Life Years) using 2018 data. In the second stage, the bias corrected technical inefficiency scores were regressed against 14 exogenous factors using a bootstrapped truncated regression.

**Results:**

The mean bias-corrected technical efficiency score of the 47 counties was 69.72% (95% CI 66.41–73.01%), indicating that on average, county health systems could increase their outputs by 30.28% at the same level of inputs. County technical efficiency scores ranged from 42.69% (95% CI 38.11–45.26%) to 91.99% (95% CI 83.78–98.95%). Higher HIV prevalence was associated with greater technical inefficiency of county health systems, while higher population density, county absorption of development budgets, and quality of care provided by healthcare facilities were associated with lower county health system inefficiency.

**Conclusions:**

The findings from this analysis highlight the need for county health departments to consider ways to improve the efficiency of county health systems. Approaches could include prioritizing resources to interventions that will reduce high chronic disease burden, filling structural quality gaps, implementing interventions to improve process quality, identifying the challenges to absorption rates and reforming public finance management systems to enhance their efficiency.

**Supplementary Information:**

The online version contains supplementary material available at 10.1186/s12962-021-00332-1.

## Background

Kenya, like many other low- and middle-income countries, has made a commitment to achieve universal health coverage (UHC) [1]. UHC means that everyone has access to good quality healthcare services that they need without experiencing financial hardship [2]. However, Kenya’s UHC aspiration is impeded by, among others, low public financing of healthcare. For instance, Kenya’s public expenditure on health is 2.3% of the country’s gross domestic product (GDP), against the recommended level of 5% required to achieve UHC [3].

In parallel with Kenya’s UHC push, the country decentralized its governance arrangements in 2013, with the formation of two tiers of government: a national government and 47 semi-autonomous county governments [4]. Decentralization refers to the transfer of functions, authority, and power from the central government to local authorities [5]. It has been promoted as a key reform for improving the provision of public services. A common typology categorizes decentralization into four main categories: (1) deconcentration which is the transfer of authority to local or regional authorities, appointed by the central authority such as districts; (2) devolution which refers to when authority is transferred to elected autonomous or semi-autonomous municipal, provincial or state governments; (3) delegation, where new powers are granted to semi-autonomous agencies established by the central authority; and (4) privatization, where private entities assume ownership of previously public entities [5]. Kenya’s version of decentralization is devolution. Within the health sector, decentralization, and more specifically devolution entailed the transfer of ownership and management of county healthcare facilities (county hospitals, health centers and dispensaries) and healthcare service delivery to the county level, while the central Ministry of Health retained the management of national referral hospitals, health policy and regulatory functions [6, 7]. Kenyan counties receive block grants from the central government, and in addition collect revenues locally, and have absolute control over their budgets and priorities [8]. They allocate funds to service areas and units, including public health facilities, based on their priorities. The relative performance of county health systems can therefore be attributed to their capacity to efficiently and effectively allocate and use available resources.

Achieving UHC in Kenya will require additional funding, that will only be justified if current resources are used efficiently. Further, improving health system efficiency has been identified as one of the key sources of fiscal space for health [9, 10]. Efficiency refers to the extent to which system objectives are met given the resources invested in the system. Two types of efficiency, technical and allocative efficiency, have been distinguished [11]. Technical efficiency is achieved when resources are allocated such that outputs are maximized for a given level of inputs, or inputs are minimized for a given level of outputs [12]. Allocative efficiency is achieved when resources are allocated such that outputs are maximized for a given level of input cost, or input costs are minimized for a given level of outputs [12].

Given the critical role that efficiency plays in improving the use of available resources and unlocking additional resources, understanding the level of health system efficiency and associated factors is an important research and policy question. Several studies have examined the technical efficiency of health systems at the national and sub-national level using data envelopment analysis (DEA) in Africa. For instance, Ngobeni et al. used DEA to assess the technical efficiency of provincial health systems in South Africa [13], Achoki et al. assessed the technical efficiency of districts in Zambia [14], while Anselmi et al. assessed technical efficiency of district health systems in Mozambique [15]. Several other papers used DEA to assess the technical efficiency of health facilities in African countries. In Kenya, In Kenya, the limited available literature shows variation in the efficiency of healthcare facilities [16–18]. There is no evidence on efficiency (and factors associated with it) at the system, rather than health facility level, in Kenya. Specifically, there is no evidence on the level and determinants of technical efficiency of county health systems using the DEA methodology in Kenya. Examining the efficiency of county health systems in Kenya is important given their central role in service provision and significant resource consumption. For instance, counties consumed 60% of the total government budget for health in the fiscal year 2015–2016 [19]. In this paper we seek to evaluate the technical efficiency of the 47 county health systems in Kenya. We elected to analyse technical rather than allocative efficiency since the later would need data on input prices which is unavailable.

## Methods

### Study setting

Kenya’s healthcare system is pluralistic, with service provision provided by both public and private healthcare facilities in almost equal measure. The public healthcare delivery system is organised into four tiers, namely community (comprising of community units), primary care (comprising of dispensaries and health centers), county referral (comprising of first and second referral hospitals) and national referral (comprising of tertiary care hospitals) [1]. The health system is financed by revenues collected by [20]:

- The government (national and county) through taxes and donor funding.
- The National Hospital Insurance Fund (NHIF) through member contributions.
- Private health insurance companies through member contributions, and
- Out of pocket spending by citizens at points of care.

Revenues mobilized by counties are allocated by the county to the various sectors, including health, in line with a programme budgeting approach. Purchasing of healthcare services is carried out through: (a) supply-side subsidies to public facilities by national and county governments; for instance, the county departments of health provides budgets to county hospitals to finance service delivery to citizens within the county, (b) the NHIF, which contracts public and private healthcare facilities in Kenya and pays them for services provided to its enrolled members, and (c) private health insurance companies that contract private healthcare facilities and pays them for services provided to their enrolled members [21]. The Kenyan health system is dependent on donor funding and out of pocket payments, with the two contributing 19.1% and 23.3% of total health expenditure, respectively according to the most recent national health accounts [22].

### Study design

This was a cross-sectional study with data collated for the year 2018 since this is the latest year where data are available. We used a two-stage double bootstrap data envelopment analysis (DEA) approach to determine the technical efficiency of county health systems and the factors that are associated with the level of county health system technical efficiency. DEA is a non-parametric linear programming method that was developed by Charnes et al. [23] to assess the relative efficiency of production units, labelled decision making units (DMU). This technique has also been employed in the health sector to assess the relative efficiency of hospitals, primary healthcare facilities, and regional health systems such as districts [24–27]. To compute the relative efficiency of a DMU, the DEA assigns weights to a set of inputs and outputs so as to maximize the efficiency score of each DMU [23, 28]. Efficiency in data envelopment analysis (DEA) is defined as the ratio of weighted sum of outputs divided by the weighted sum of inputs [23]. The DEA technique is considered particularly relevant in the health sector given the complex nature of health systems where multiple inputs are utilized to produce multiple outputs [29–31].

The first stage of the analysis estimated bootstrapped bias corrected technical inefficiency scores of each of the 47 county health systems using the DEA methodology. The second stage employed bootstrap truncated regression to regressed these technical inefficiency scores on a set of exogenous variables to identify determinants of county health system technical inefficiency.

### Input, output and exogenous variables

We obtained available data through different sources including literature review of over 100 publications on health system efficiency [32], engagement of Kenyan policy makers in a workshop [33], and data available from national and county databases, surveys and statistics. We used the following three input variables:

1. County public health expenditure (Kenya shillings).
2. County private health expenditure (Kenya shillings).
3. County number of healthcare facilities (public, private for profit, and faith-based facilities).

These variables comprehensively capture the inputs into a county health system: county public health expenditure captures all public inputs (expenditures on staff, healthcare commodities, and operations and maintenance costs) for both health facility based and non-health facility based health activities, while private health expenditure captures inputs by the private sector. However, these two sets of inputs do not comprehensively capture capital inputs. We therefore also included the number of healthcare facilities as an input to represent capital inputs. These input data were divided by 1,00,000 population size for each county using population data from the Kenya National Bureau of statistics to obtain per capita values [34].

We used estimates of disability adjusted life years (DALYs) per county to represent health outcome of the production process. Specifically, we obtained data on DALYs per 1,00,000 population per county from the global burden of disease study modelled estimates for Kenya [35] and computed their reciprocal, such that a higher value (of this computed reciprocal) reflects better health outcomes, i.e., a lower burden of disease. This is because the DEA methodology assumes that the outputs in the production process are desirable [36]. We have divided all the inputs and outputs by 1,00,000 population to standardize the input and output variables, which is one of suggested method for addressing the potential problem of ratio variables in DEA [37].

Variation in disease burden across counties will be affected by other underlaying factors such as demographic characteristics. These factors are accounted for in the exogenous variables in the second stage of the analysis. We used a range of exogenous variables drawn from demographic, disease burden, health risk, socio-economic, and health system determinants of health. Table 1 outlines the variables used in the DEA model.

**Table 1:** Description of input, output and exogenous variables

Variable	Description	Data source
Output variable		
DALYS	The reciprocal of DALY rates per 1,00,000 population 2018	Global burden of disease
Inputs		
County public health expenditure (Kenya shillings)	County public health expenditure per 1,00,000 population 2018	Controller of budget reports
County private health expenditure	County private health expenditure per 1,00,000 population 2018	KHHEUS
Number of healthcare facilities (Kenya shillings)	Number of healthcare facilities (public and private) in the county per unit population 2018	KHFS
Exogenous variables		
Under 5 years population	Proportion of the population under 5 years	KHIBS survey
Elderly population	Proportion of the population over 60 years	KHIBS survey
Level of corruption	County corruption index	EACC ethics and corruption survey
HIV burden	The prevalence of HIV in the county	Kenya aids indicator survey
Development budget absorption	The proportion of the annual county development budget that is executed (spent/implemented)	Controller of budget reports
Recurrent budget absorption	The proportion of the annual county recurrent budget that is executed (spent/implemented)	Controller of budget reports
Alcohol consumption	Proportion of the population taking alcohol	STEPS survey
Level of literacy	Proportion of the population that is literate	KDHS
Quality of care	Quality of care index	SDI
Population density	Population density per square kilometres	Kenya census data
Urbanization	Proportion of population living in the urban areas	Kenya census data
County economic performance	County per capita GDP	CRA report
Private	Private health facilities as a proportion of all health facilities in the county	KHHEUS
Absenteeism	Proportion of absenteeism of health workers	SDI
Autonomy	Full autonomy-public health facilities have complete access to and flexibility to spend funds	County informants
	Partial autonomy—public health facilities have access to some funds but not others	
	No autonomy—public health facilities have no access to any funds, but rather redirect them to the county department of health	
Water	Proportion of households that with clean water	KHIBS survey
Smoking	Proportion of the population that smokes	STEPS survey

#### DEA estimation of technical inefficiency scores

The original non-linear model is shown in Eq. 1.

1 $$Max h = \frac{{\mathop \sum \nolimits_{r = 1}^{s} u_{r} y_{{rj_{o} }} }}{{\mathop \sum \nolimits_{i = 1}^{m} v_{i} x_{{ij_{o} }} }}$$

Subject to: Eq. (1)

$$\frac{{\mathop \sum \nolimits_{r = 1}^{s} u_{r} y_{rj} }}{{\mathop \sum \nolimits_{i = 1}^{m} v_{i} x_{ij} }} \le 1$$

*u*_*r*_, *v*_*i*_ ≥ 0.

where *h* is the relative efficiency of DMU, *y*_*rj*_ is the amount of output r produced by county j, *x*_*ij*_ is the amount of input i used by county j, *u*_*r*_ is the weight given to output *r*, *v*_*i*_ is the weight given to input *i*, *j*  = 1,…,*n;* n is the number of counties, *r*  = 1,…,*s*; s is the number of outputs, *i*  = 1,…,*m*; m is the number of inputs.

The procedure of assessing efficiency in DEA involves solving linear programming tasks for each of the DMUs under evaluation and the non-linear model can be converted to a linear model for the output-oriented model by letting $\mathop \sum \nolimits_{r = 1}^{s} u_{r} y_{rj} = 1$ as shown in Eq. 2. In this case, the efficiency score is equal to 1/h_o_.

Min $${h}_{o}$$ = $$\mathop \sum \limits_{i = 1}^{m} v_{i} x_{{ij_{o} }}$$.

Subject to:

2 $$\mathop \sum \limits_{r = 1}^{s} u_{r} y_{{rj_{o} }} = 1$$

$$\mathop \sum \limits_{i = 1}^{m} v_{i} x_{ij} - \mathop \sum \limits_{r = 1}^{s} u_{r} y_{rj} \ge 0$$

where *j*  = 1,…,n.

*u*_*r*_, *v*_*i*_ ≥ ε, with ε > 0, where ε is non archimedian.

r  = 1,…s; i  = 1,…m.

DMUs receive scores between 0 (0%) (least efficient) and 1 (100%) (most efficient) with the efficient DMUs forming a production frontier that envelopes others, and to which all inefficient DMUs are compared. We designated a county health system as the DMU and analysed all 47 counties in Kenya.

The DEA method has several limitations. First, DEA results may be influenced by measurement error or statistical noise: since the DEA methodology is non-stochastic, it ascribes deviations from the frontier entirely to inefficiency, even though these may be due to measurement errors [38]. This weakness is addressed by the use of a model that corrects for measurement error. Second, DEA does not produce estimates that can be validated using conventional statistical methods. We applied a bootstrapping approach to allow statistical inference methods that can be used to generate confidence intervals. Third, since DEA results are dependent on the input–output mix, the exclusion of an important input or output may result in bias. We addressed this by seeking to select variables that comprehensively captures inputs and outputs to a county health system. Fourth, when there are few observations and many inputs and/or outputs, many DMUs will appear to be at the frontier, overstating their efficiency. We used the entire universe [47] of counties as DMUs which is addresses this potential limitation. Fifth, treating inputs and/or outputs as homogeneous commodities when they are heterogeneous may result in bias. Two of our 3 input variables are comparable (i.e., they are both expenditures) and we use only one output variable, thus minimizing this potential bias. Sixth, DEA efficiency scores are measured relative to the best practice within selected sample of DMUs [38]. Usually, there exists more than one efficient unit and these scores cannot be further compared directly to each other purely based on efficiency scores. Although there are suggested methods and models such as ranking that can be used to mitigate this challenge, we, did not directly compare efficiency scores in this study[39].

### Stepwise regression

For the selection of exogenous variables to be included in the analysis, we regressed a set of exogenous variables on the technical inefficiency scores. These factors are characteristics of the county or of its environment and are either actionable ones (the county can do something about it) or descriptors of the diverse situations that counties face. Starting from a long list of potential factors from different sources, we dropped those for which (a) data were not available, (b) there were correlations with inputs (p value  < 0.05), and (c) there was no statistical relationship between the variable and the inefficiency score at the 10% significance level using a stepwise selection approach (i.e., regressing the inefficiency scores with each of the variables individually to assess the bivariate relationship). We checked the distributions of each of the variables and used suitable transformations for non-normally distributed variables. Table 2 outlines the characteristics of the variables and transformations used. Variables that were normally distributed were not transformed.

**Table 2:** Descriptive characteristics of model variables

Variable	Mean (95% CI)	Median (min., max.)	Data transformation
Output variable			
DALYS	0.23 (0.22–0.25)	0.23 (0.15–0.42)	No transformation
Outputs			
County public health expenditure	2114.40 (1795.23–2433.58)	2018 (326–6018)	No transformation
County private health expenditure	2310.83 (2079.90–2541.76)	2,156 (814–4242)	No transformation
Number of healthcare facilities	2.42 (2.17–2.67)	2.3 (1–4.9)	No transformation
Exogenous variables			
Level of corruption	1.67 (1.44–1.90)	1.52 (1–5.53)	Square root
HIV burden	4.45% (3.14–5.76%)	3.50% (0.10–21.00%)	Logit
Development budget absorption	98.09% (83.74–112.45%)	97.70% (38.80–353.60%)	Logit
Recurrent budget absorption	95.21% (88.95–101.47%)	98.60% (20.50–166.30%)	Logit
Alcohol consumption	20.83% (17.98–23.68%)	20% (0–40%)	No transformation
Level of literacy	91.38% (88.11–94.66%)	96.40% (53.80–99.70%)	Logit
Quality of care	75.70% (73.91–77.48%)	74.70% (62.00–91.20%)	No transformation
Population density	509.15 (164.03–854.27)	221 (6–6247)	Log
County economic performance	144,146.6 (121,684.4–166,608.9)	135,135.50 (40,464.25–384,156.7)	Log
Private	41.55% (36.56–46.55%)	37% (2–80%)	No transformation
Absenteeism	51.51% (48.95–54.08%)	50.30% (24.90–67.60%)	No transformation
Water	65.33% (60.06–70.59%)	67.20% (27.80–97.10%)	No transformation
Smoking	8.53% (6.93–10.13%)	9% (0–40%)	No transformation
Autonomy			
Full autonomy—10 counties			
Partial autonomy—9 counties			
No autonomy—28 counties			

### Data analysis and model specification

We used the Simar-Wilson double bootstrap model to carry out the analysis. We selected the Simar-Wilson two-stage double bootstrap procedure because it corrects for measurement error and serial correlation (estimated DEA scores are from a common sample of data and hence are not independent), both limitations that are associated with DEA. The Simar-Wilson model derives bootstrapped DEA inefficiency scores in the first stage and carries out a bootstrapped truncated regression of these bias-corrected inefficiency scores on exogenous variables. In this study, we used a variable-returns to scale (VRS), output oriented DEA model. We chose the VRS model based on the assumption that not all counties were operating at their optimal scale and that there would be economies and diseconomies of scale. We chose an output-oriented model based on the knowledge that counties in Kenya have a relatively fixed quantity of inputs and hence managers have more leeway in controlling outputs rather than inputs. We calculated mean, standard deviation and 95% confidence intervals for the efficiency levels. An exogeneous variable was considered significant if the p-value was less than 0.05. Data analysis was carried out in STATA 14 software [40] and R statistical programming software [41].

## Results

### Efficiency of county health systems

**Fig. 1:**
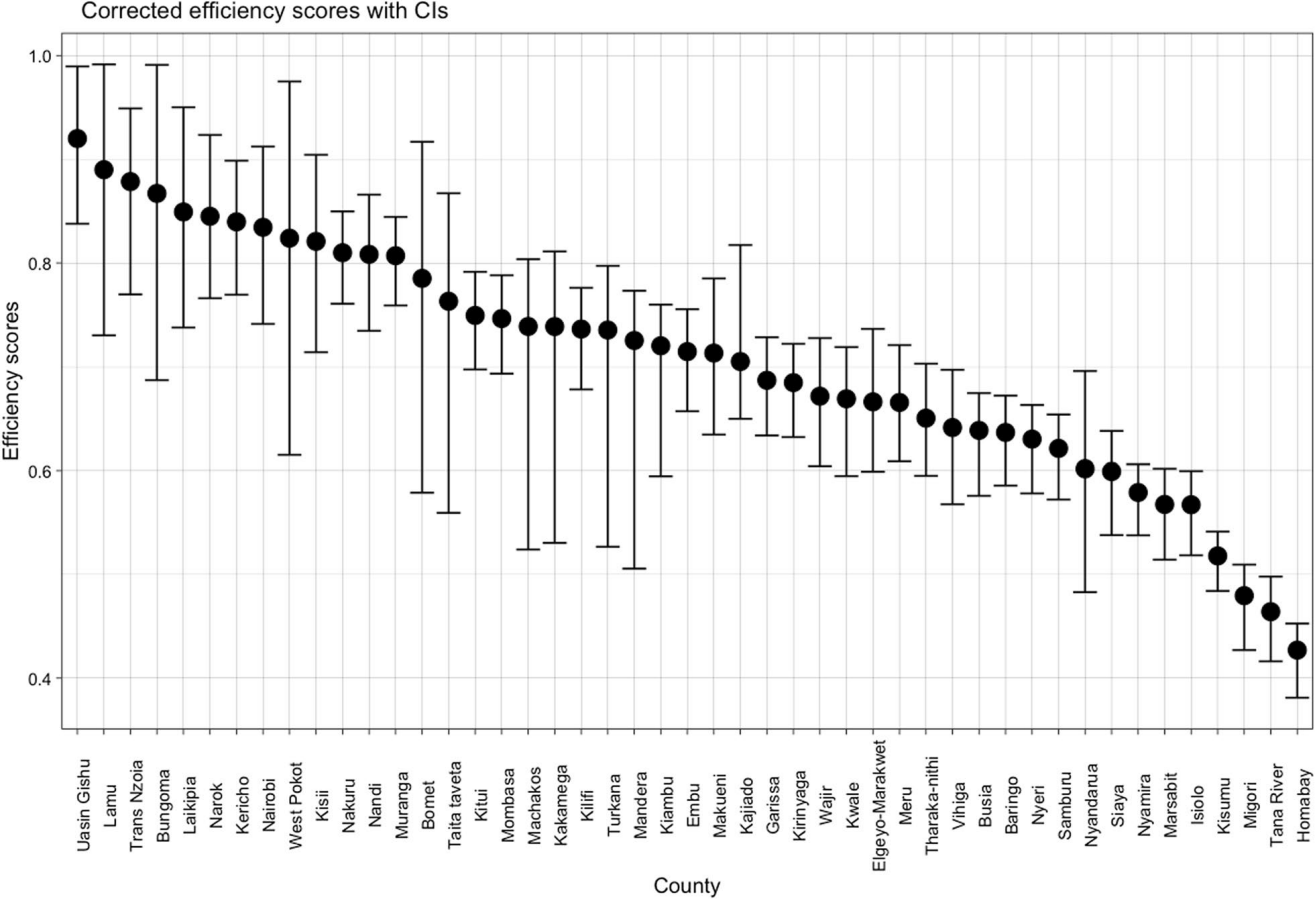
County bias-corrected technical efficiency scores

To present technical efficiency scores, we computed the reciprocal of the technical inefficiency scores generated in the first stage of the DEA, since this is more intuitive to present and interpret. Figure 1 shows the bootstrapped technical efficiency score of each of the 47 county health systems with their respective confidence intervals. The mean technical efficiency score of county health systems in Kenya is 69.72% (95% CI 66.41–73.01%), indicating that on average, county health systems could increase their outputs by 30.28% at the same level of inputs. County technical efficiency scores range from 42.69% (95% CI 38.11–45.26%) for Homabay county to 91.99% (95% CI 83.78–98.95%) for Uasin Gishu county.

### Factors associated with county health system technical inefficiency

Findings from the bias-corrected Simar-Wilson regression reveal that four variables have a significant association with the county health system technical inefficiency scores (Table 3). These are (a) HIV prevalence (b) population density (c) quality of care index and (d) development budget absorption. On the one hand, higher HIV prevalence is associated with greater technical inefficiency of county health systems. On the other hand, a high population density, higher county absorption of development budgets, and higher quality of care provided by healthcare facilities are associated with lower county health system inefficiency.

**Table 3:** Associations of Simar-Wilson bias-corrected inefficiency scores

Variable	Regression coefficient	Bootstrap standard errors	95% CI	P Value
Level of corruption	− 0.089	0.170	(− 0.426, 0.240)	0.600
HIV burden	0.120	**0.049**	(0.022, 0.214)	**0.014**
Development budget absorption	− 0.031	**0.013**	(− 0.055, − 0.005)	**0.013**
Recurrent budget absorption	0.008	0.018	(− 0.028, 0.041)	0.635
Alcohol consumption	− 0.008	0.045	(− 0.080, 0.01)	0.069
Level of literacy	0.039	0.061	(− 0.080, 0.156)	0.52
Quality of care	− 0.017	**0.007**	(− 0.029, − -0.002)	**0.021**
Population density	− 0.100	**0.045**	(− 0.186, − 0.012)	**0.024**
County economic performance	− 0.046	0.095	(− 0.234, 0.140)	0.633
Private	0.003	0.003	(− 0.003, 0.010)	0.322
Absenteeism	− 0.005	0.005	(− 0.014, − 0.004)	0.300
Autonomy	0.034	0.005	(− 0.055, 0.120)	0.447
Water	0.00	0.003	(− 0.005, 0.005)	0.942
Smoking	− 0.013	0.009	(− 0.029, 0.005)	0.127

## Discussion

In this paper, we have presented results of an analysis of the technical efficiency of country health systems in Kenya. Our analysis finds that there is considerable scope for county health systems to improve their outcomes though efficiency gains. On average, counties could increase their outcomes by 30% with available resources. There is however substantial heterogeneity across counties. For instance, 3 counties, and 4 counties have technical efficiency scores of below 50% and 60% respectively and hence could increase their outputs by between 40 and 50% with existing resources, and eight counties have technical efficiency scores greater than 80%. These findings highlight the fact that county health systems are characterized by substantial technical inefficiency. This level of inefficiency is comparable to that found in other settings. For instance, an analysis of the efficiency of health systems in 45 Sub-Saharan African countries reported a mean efficiency score of 80% implying a 20% level of resource wastage [42], while an analysis of the efficiency of state health systems in India reported mean efficiency scores of between 54.4% and 65.7% over a 5 year period, implying resource wastage levels of between 34.3 and 45.6% [43].

The second stage regression of bias corrected inefficiency scores reveal that higher HIV prevalence was associated with greater technical inefficiency of county health systems, while higher population density, county absorption of development budgets, and quality of care provided by healthcare facilities are associated with lower county health system inefficiency. The positive association between a high burden of HIV and health system inefficiency is explained by the fact that a high disease burden worsens population health outcomes and may also consume greater healthcare resources compared to regions with low disease burden. In Kenya, while the mean HIV prevalence is 4.5%, the counties with the highest HIV prevalence (Homabay 21%, Siaya 21%, Kisumu 16%, Migori 13%) are among the counties with the lowest technical efficiency scores. This finding reflects that of an assessment of the technical efficiency of African country health systems that found that a high HIV burden was associated with higher country health system technical inefficiency [42]. Beyond HIV, other studies have found the burden of chronic diseases more broadly is negatively associated with the technical efficiency of health systems. For instance a 10% increase in the proportion of people with chronic conditions was associated with a 10–18% increase in technical inefficiency scores of regional health systems in Canada [44].

Literature reports mixed findings on the association between population density and health system efficiency. Our study reports similar findings to those of a study on Chile that found that a high population density of primary healthcare catchment areas reduced the technical inefficiency of regional health systems [45]. It has been argued that a higher population density reduced the technical inefficiency of regional health systems by reducing per capita cost of healthcare [46]. However, a study of Finnish municipalities found that a high population density increased the technical inefficiency of municipalities and speculated that this could be because high population densities could compromise quality of care [47].

The negative association between county development budget absorption and technical inefficiency is explained by the fact that a high capacity to absorb budgets unlocks resources that may contribute to improving health systems outcomes. Low budget absorption reduces the amount of resources that are available to be invested in health inputs, which in turn reduces the capacity of the health system to produce heath outputs and outcomes. Public finance management in the health sector, including the capacity of the health sector to absorb has been shown to affect the efficiency of health systems [48]. For instance, rigid public finance management (PFM) controls were shown to increase inefficiency of health systems in Tanzania and Zambia by limiting the flexibility of budget execution [48].

The observation that quality of care is negatively associated with technical inefficiency is explained by the fact that improved quality of care improves health outcomes. Quality of care has been identified as one of key determinants of health system efficiency in several ways including that poor quality of care increases wastage due to unnecessary care and compromises health outcomes [10].

The findings from this analysis highlight the need for county health departments to consider ways to improve the efficiency of county health systems. For counties with high HIV burden, it is imperative that they prioritize resources to interventions that will reduce this burden. The data used in this analysis (Additional file 1) shows that the counties with the highest HIV burden all have below average, and among the lowest, per capita public health expenditures. This could indicate the need for greater investments in the health sector in high disease burden counties. Interventions to improve the quality of care provided by healthcare facilities are also a lever that should be used to enhance the efficiency of county health systems. Previous assessments have revealed gaps in structural capacity including inadequate number of health workers, poor availability of essential health commodities and equipment [49], and in poor process of care [50]. Studies have also shown that mechanisms to monitor quality of care and hold health facilities in Kenya to account are inadequate [21, 51]. Counties should focus on filling these structural gaps and implementing interventions to improve process quality. The PFM challenges faced by county governments in Kenya, including budget absorption, have been identified [52, 53]. This have included delayed disbursement of funds to counties by the national government and lengthy and rigid procurement processes that together reduce the capacity of county governments to spend allocated budgets [52, 53]. It is imperative that counties reform PFM systems to enhance their efficiencies.

In addition to the generic limitations of the DEA approach outlined in the methods section, we highlight here four additional limitations. First, we used modelled data on DALYs from the 2019 Global burden of disease study, which are unlikely to be as accurate as locally collected data. However, local data on burden of disease at the county level is not available and so the modelled estimates are the only data available. Second, DALYs are an undesirable outcome and yet DEA is designed for desirable outcomes. This necessitated the transformation of DALY variable to its reciprocal, which creates a problem of ratio variables. It has been observed ratio variables violates the convexity assumptions of DEA [54]. To address this, we chose the approach to standardize inputs and outputs by dividing by the same denominator (1,00,000 population) as proposed by Sopko and Kucisova [37]. We appreciate that while other methods have been proposed to address this challenge, there is no consensus in the literature on a preferred approach. Third, in comparing the relative efficiency of counties, it is likely that we have not fully accounted for important structural and organization factors that play a key role as determinants of service delivery. For instance, some of the factors that Kenyan health sector stakeholders identified as potentially affecting country health system efficiency could not be captured quantitatively. These factors include managerial capacity, political influence, and coordination of actors [33]. This weakness highlights the need for mixed methods approaches to efficiency analysis to facilitate the documenting of factors that might not be quantitatively measured. Indeed, this is the approach that we adopted for this study, which is part of a larger mixed methods study that has documented and reported qualitative findings elsewhere [33]. Fourthly, since we did not report the traditional DEA scores, we did not also report on peer analysis and targets that each county needs to achieve in order to achieve efficiency.

## Conclusion

To improve the performance of county health systems, county health decision makers need information about how well their counties are utilizing the resources available to them. Our findings show that county health systems in Kenya are associated with substantial inefficiency, signalling considerable wastage of resources. The analysis reveals that determinants of county health system efficiency includes HIV prevalence, population density, absorption of development budgets, and quality of care. The findings signal a need to implement interventions to achieve efficiency gains, especially in the context of a tension between a commitment to achieve UHC and scarce resources. Further work is required to explore the mechanisms by which identified determinants affect the efficiency of health systems to identify policy levers for efficiency gains.

## Supplementary Information

**Additional file 1.** Data used for the efficiency analysis of county health systems in Kenya.

## Data Availability

All the data used for this study is publicly available as Additional file.

## Abbreviations

AE: Allocative efficiency
DALYs: Disability adjusted life years
DEA: Data envelopment analysis
DMU: Decision making unit
GDP: Gross domestic product
HIV: Human immunodeficiency virus
LMICs: Low and middle income countries
NHIF: National hospital insurance fund
PFM: Public finance management
TE: Technical efficiency
UHC: Universal health coverage
